# Genetic analysis of children with suspected immunodeficiency: mimickers of inborn errors of immunity

**DOI:** 10.1007/s00431-026-06778-w

**Published:** 2026-03-05

**Authors:** Saniye Yasemin Yılmaz, İlknur Külhaş Çelik, Ebru Marzioğlu Özdemir, Hasibe Artaç

**Affiliations:** 1 Department of Pediatric Immunology and Allergy, Selcuk University Faculty of Medicine, Konya, Turkey; 2 Department of Medical Genetics, Selcuk University Faculty of Medicine, Konya, Turkey

**Keywords:** Inborn errors of immunity, Genetic testing, Whole-exome sequencing, Mimickers of immunodeficiency

## Abstract

Inborn errors of immunity (IEI) comprise a heterogeneous group of disorders with diverse clinical manifestations. In this study, we aimed to evaluate genetic findings in patients with suspected IEI and to assess the contribution of next-generation sequencing (NGS) in identifying both IEI-related and non-IEI-related genetic variants. Between January 2020 and January 2025, 91 pediatric patients (0–18 years) referred for suspected IEI were retrospectively analyzed. Demographic data, clinical features, immunological profiles, and genetic results were reviewed, including single-gene sequencing, fluorescence in situ hybridization (FISH), targeted gene panels (TGP), and whole-exome sequencing (WES). Patients analyzed by NGS were classified into three categories according to detected variants: IEI-related, non-IEI-related, and undetected disease-causing variant. A total of 79 patients underwent NGS-based genetic testing. The mean age was 4.37 ± 5.09 years. WES was performed in 40 patients (50.6%) and TGP in 39 (49.4%). Pathogenic variants linked to IEI-related were detected in 28 patients (35.4%), whereas non-IEI-related pathogenic variants were identified in 12 (15.2%). The remaining 39 patients (49.4%) had undetected disease-causing variants. The diagnoses of patients carrying pathogenic variants unrelated to IEI included primary ciliary dyskinesia, Ellis–van Creveld syndrome, desmoglein-1 deficiency, and others.

*Conclusion*: Our study highlights the importance of genetic testing in the differential diagnosis of IEI and provides evidence supporting its role in identifying mixed IEI phenotypes. Comprehensive interpretation of genetic results within a multidisciplinary clinical framework is essential for accurate diagnosis, appropriate management, and effective genetic counseling.

**What is Known:**

• *Inborn errors of immunity are rare, genetically heterogeneous disorders with overlapping phenotypes, often causing delayed diagnosis and necessitating next-generation sequencing*.

• *Recent multicenter studies show that whole-exome sequencing has increased diagnostic yield to over 40-50% and improved clinical classification and management*.

**What is New:**

• *Comprehensive genetic testing revealed clinically significant non–IEI-related variants in 15.2% of patients with mixed or atypical IEI phenotypes*.

• *A multidisciplinary approach broadened the diagnostic perspective beyond classical IEI, enabling more accurate and individualized patient management*.

## Introduction

Inborn errors of immunity (IEI) are a heterogeneous group of genetic disorders that impair one or more components of the immune system. They are characterized by increased susceptibility to infections, autoimmunity, malignancies, and immune dysregulation, and their rarity often leads to delayed or missed diagnoses [1]. The International Union of Immunological Societies (IUIS) regularly updates the list of monogenic IEIs as more genetic defects are identified [2]. Although patient history, clinical features, and laboratory findings may suggest certain IEI types, the wide range of genes causing overlapping phenotypes makes diagnosis challenging. Therefore, next-generation sequencing (NGS), which enables simultaneous analysis of multiple causative genes, has become an essential diagnostic tool.

In patients undergoing genetic testing for suspected IEI, clinically relevant mutations in non-IEI-related genes can substantially affect diagnosis, management, and genetic counseling. For instance, a Brazilian study identified pathogenic variants in genes such as *ABCA12* and *SLC25A13*—genes unrelated to immune function—in individuals evaluated for IEI. These findings indicate that patients’ symptoms may not be solely attributable to IEI and that alternative or concomitant genetic factors may influence their clinical presentation [3].

Advances in molecular genetics—especially NGS-based methods—have transformed IEI diagnosis, enabling genetic identification even in complex or atypical cases [4, 5]. The widespread use of genetic testing has not only facilitated definitive diagnosis but also enabled tailored therapeutic strategies and accurate genetic counseling [6]. In recent years, novel NGS techniques such as whole-exome sequencing (WES) have further improved the diagnostic yield in patients with suspected IEI. In a large multicenter study of 278 families from 22 countries, Stray-Pedersen et al. [7] showed that WES provided a definitive diagnosis in 40% of cases, led to clinical reclassification in nearly half, and directly influenced management in one-fourth of patients.

## Methods

This retrospective study included 91 patients aged 0–18 years who were evaluated between January 2020 and January 2025 in the Department of Pediatric Immunology and Allergy and who underwent genetic testing for a preliminary diagnosis of primary immunodeficiency. Medical records were reviewed retrospectively. The results of single-gene sequencing, fluorescence in situ hybridization (FISH), and NGS (targeted gene panel [TGP] or WES) were recorded. All patients diagnosed with IEI according to the criteria of the European Society for Immunodeficiencies Working Group were classified based on the IUIS’s phenotypic classification [2].

Chromosomal analysis was performed in patients with dysmorphic features, and FISH testing was used when DiGeorge syndrome was suspected. Single-gene sequencing was applied in the presence of a relevant family history, while NGS was used for patients with immunodeficiency-related clinical features. NGS results were classified into three groups: IEI-related variants, non-IEI-related variants, and undetected disease-causing variant. Phenotypic and genotypic findings were then compared across these categories.

According to the clinical presentation, complete blood count, serum immunoglobulin (Ig) levels (IgG, IgA, IgM, and IgE), and lymphocyte immunophenotyping (CD3, CD4, CD8, CD19, and CD16/56) were recorded. The dihydrorhodamine test was performed to assess phagocytic function in patients with relevant clinical suspicion. Echocardiography was conducted when cardiac pathology was suspected. Most variants identified in our cohort have been previously reported in public databases, including ClinVar and *Human Gene Mutation Database* (HGMD), and their classification was based on existing evidence [8]. For variants without prior reports, classification was performed according to American College of Medical Genetics and Genomics/Association for Molecular Pathology (ACMG/AMP) guidelines, incorporating population frequency data, in silico prediction tools, segregation information when available, and detailed phenotype correlation [9]. Importantly, variants without sufficient evidence for pathogenicity were classified as variants of uncertain significance (VUS) and were not considered definitive molecular diagnoses. Novel or rare variants are interpreted cautiously within the context of clinical and immunological findings, rather than being presented as pathogenic mutations in isolation. A standardized data collection form was used. The study was approved by the Ethics Committee of Selçuk University Faculty of Medicine (approval number 2025/67; meeting date February 11, 2025). Written informed consent for the use of clinical and genetic data was obtained from the patients’ legal guardians.

Data entry and statistical analyses were performed using SPSS for Windows, version 18.0 (SPSS Inc., Chicago, IL, USA). The normality of distribution was assessed using visual methods (histograms and probability plots) and analytical tests (Kolmogorov–Smirnov and Shapiro–Wilk tests). Numerical variables were summarized as medians (interquartile ranges IQRs), and categorical variables were presented as frequencies and percentages. Comparisons between nonnormally distributed numerical variables and categorical data were performed using the Mann–Whitney *U* test. Categorical variables were analyzed with the chi-square (*χ*^2^) test. When more than 20% of expected cell counts were below the minimum expected frequency (*n* = 5), categories were combined; if this was impossible, the Fisher’s exact test was used. A *p*-value < 0.05 was considered statistically significant.

## Results

**Fig. 1:**
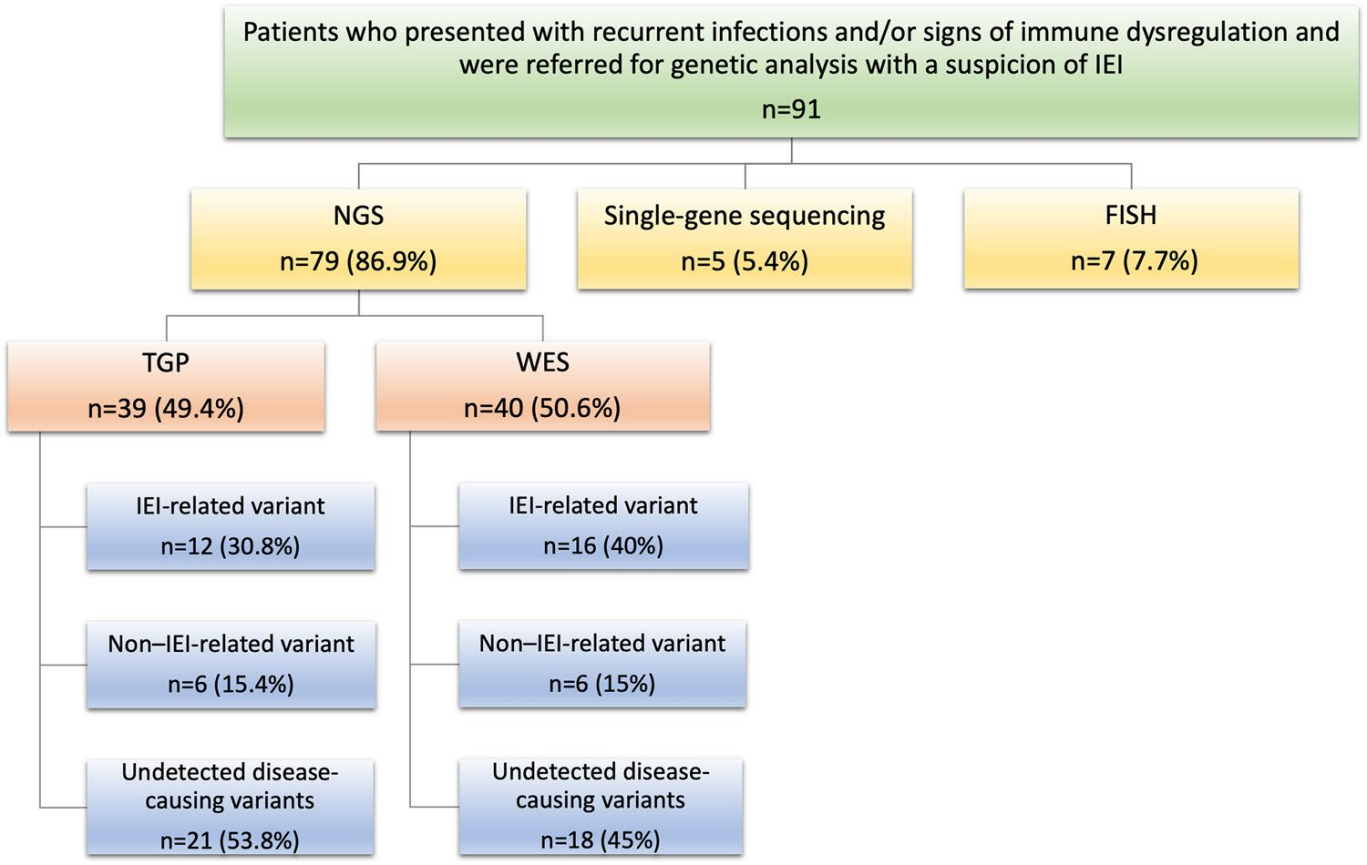
Genetic test methods in patients with suspicion of IEI (Abbreviations: FISH: fluorescence in situ hybridization, IEI: inborn errors of immunity, NGS: next-generation sequencing, TGP: targeted gene panels, WES: whole-exome sequencing)

This study included 91 patients (43 females, 48 males) who underwent genetic testing. As shown in Fig. 1, FISH was performed in 7.7% of cases and single-gene sequencing in 5.4%. FISH identified 22q11 deletions in seven patients. Among the five patients who underwent single-gene sequencing, four had IEI-related diagnoses (two Bruton agammaglobulinemia [BTK], one purine nucleoside phosphorylase [PNP], one IL10R deficiency), while one was diagnosed with congenital sucrase–isomaltase deficiency.

**Fig. 2:**
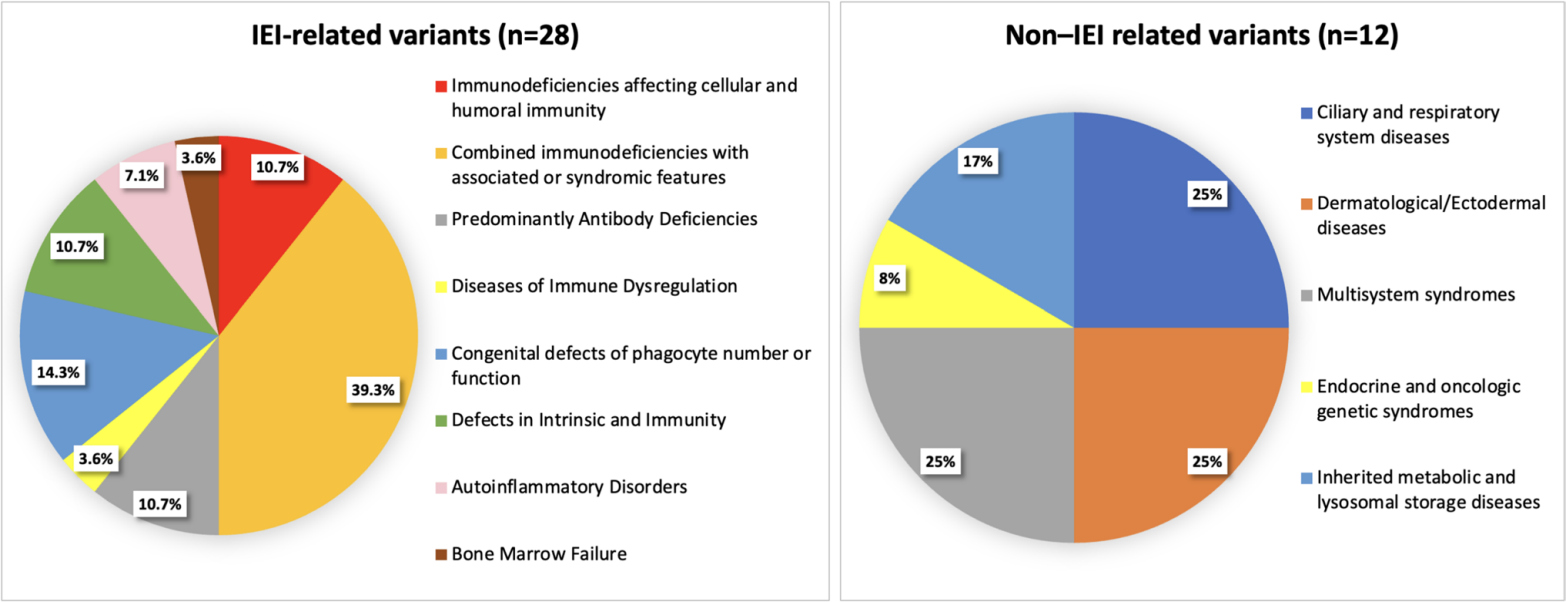
Distribution of the patients based on their clinical diagnosis (Abbreviations: IEI: inborn errors of immunity)

The remaining 79 patients were evaluated using TGP and WES. TGP testing (*n* = 39, 49.4%) was preferred for cases with a strong clinical suspicion focusing on specific genes, whereas WES (*n* = 40, 50.6%) was performed for patients presenting with complex clinical and laboratory findings. Among these 79 patients (86.8%), pathogenic variants associated with IEI were identified in 28 cases (35.4%). In addition, 12 patients (15.2%) carried clinically significant but non-IEI-related variants, while disease causing variant were not detected in the remaining 39 patients (49.4%). According to the IUIS classification, the distribution of the 28 patients with IEI-related variants detected by NGS is presented in Fig. 2. The highest proportion was observed in the group with syndromic combined immunodeficiencies, comprising 11 patients (39.3%). This was followed by defects in phagocyte number or function in four patients (14.3%), intrinsic and innate immunity defects in three patients (10.7%), antibody deficiencies in three patients (10.7%), and combined immunodeficiencies in three patients (10.7%). Lower frequencies were noted for autoinflammatory diseases in two patients (7.1%), bone marrow failure in one patient (3.6%), and diseases of immune dysregulation in one patient (3.6%).

The distribution of gene variants identified in patients who received a genetic diagnosis through NGS is presented in Fig. 2. The most frequently affected gene was *STAT3*, with pathogenic variants detected in three patients. Significant variants in *TTC37* and *PNP* genes were each identified in two patients. These genes are associated with distinct clinical entities: hyper-IgE or hypereosinophilic syndromes (*STAT3*), *TTC37*, and *PNP* deficiency. *ATM* and *CFTR* mutations were also reported in two patients each, corresponding to ataxia–telangiectasia and cystic fibrosis, respectively. Other genetic defects involved genes such as *CD27*, *CD40LG*, *CDCA7*, *DNMT3B*, *FCGR3A*, *IRF2BP2*, *MEFV*, *NCF1*, *OTULIN*, *PAX1*, *RANBP2*, *RTEL1*, *STK4*, *TNFSF13*, *BTK*, *USB1*, and *ZNFX1*.

Using NGS, non-IEI-related variants were identified in 12 patients (Fig. 2). Three patients in the ciliary and respiratory system diseases group were diagnosed with primary ciliary dyskinesia (PCD). In the Dermatological/Ectodermal diseases group, one patient had desmoglein-1 deficiency and another had premature ichthyosis syndrome. In the multisystem syndromes group, one patient each was diagnosed with Primrose syndrome, megalencephaly–capillary malformation–polymicrogyria syndrome, and Ellis–van Creveld syndrome. In the endocrine and oncologic genetic syndromes group, one patient was identified with multiple endocrine neoplasia type 2A. Additionally, in the inherited metabolic and lysosomal storage diseases group, one patient had congenital disorder of glycosylation type Ia and another had GM1 gangliosidosis.

NGS analysis identified genetic variants in 40 patients diagnosed with IEI-related or non-IEI-related conditions. According to ACMG classification, 16 variants were pathogenic and 14 were likely pathogenic. The clinical and laboratory findings of these patients were found to be consistent with the identified genetic variants. Among the 10 patients classified as having variants of VUS, three of them showed a clinical phenotype consistent with PCD. The patient with OFD1 variant had asplenia, recurrent otitis and pneumonia accompanied by situs inversus. Two patient with FOXJ1 variant and compound DNAH11 mutation had recurrent lower respiratory tract infections and bronchiectasis. The patient diagnosed with CFTR presented with recurrent lower respiratory tract infections, diarrhea, growth retardation, and a positive sweat test, which supported the diagnosis. Two patients was diagnosed as trichohepatoenteric syndrome. Overall, both patients exhibited largely overlapping clinical phenotypes, characterized by chronic diarrhea, recurrent infections, and hypogammaglobulinemia. The individual carrying an RANBP2 variant had a history of influenza-associated acute necrotizing encephalopathy, with a sibling who had previously died from the same condition. The patient with the RTEL1 variant presented with recurrent abscesses, severe neutropenia, and neuromotor developmental delay. The case with DNMT3B variant had recurrent infections, hypogammaglobulinemia, and reduced memory B-cell counts; the diagnosis was confirmed by the presence of centromeric instability. In the patient with a FCGR3A variant, flow cytometry analyses demonstrated an absence of CD16 expression in Natural Killer cells.

Demographic features of patients evaluated by TGP and WES were compared across three groups: those with IEI-related, clinically significant but non-IEI-related variants, and undetected disease-causing variants (Table 1). Consanguinity and shared village of origin were significantly more common in patients with IEI (*p* < 0.05). Among the 28 patients diagnosed with IEI by NGS, 12 (42.9%) were identified through TGP testing and 16 (57.1%) through WES. Thirteen patients (46.4%) were female and 15 (53.6%) were male. The median age at presentation was 1.48 years (IQR: 0.62–6.68), and the median age at genetic diagnosis was 3.41 years (IQR, 1.93–7.74). Age and sex distributions did not differ significantly between the IEI-related and non-IEI-related groups.

**Table 1:** Comparison of demographic characteristics of patients analyzed by TGP and WES according to variant characteristics

	IEI-related variants (*n* = 28)	Non-IEI-related variants (*n* = 12)	Undetected disease-causing variants (*n* = 39)	*p*-value
	*n* (%)	*n* (%)	*n* (%)	
Genetic diagnosis (years)/median (1st–3rd quartile)	3.41 (1.93–7.74)	1.77 (0.82–5.52)	6.28 (1.88–12.83)	0.241^a^
Age at admission (years)/median (1st–3rd quartile)	1.48 (0.62–6.68)	1.26 (0.53–2.69)	2.05 (0.58–9.67)	0.487^a^
NGS method				
* TGP*	12 (42.9)	6 (50.0)	21 (53.8)	0.674^b^
* WES*	16 (57.1)	6 (50.0)	18 (46.2)	
Sex				
* Female*	13 (46.4)	7 (58.3)	18 (46.2)	0.743^b^
* Male*	15 (53.6)	5 (41.7)	21 (53.8)	
Maturity				
* Mature*	19 (67.9)	7 (58.3)	27 (69.2)	0.546^b^
* Premature*	7 (25.0)	5 (41.7)	11 (28.2)	
* Postmature*	2 (7.1)	0 (0.0)	1 (2.6)	
Birth				
* Normal vaginal*	15 (55.6)	4 (33.3)	18 (47.4)	0.437^b^
* Cesarean section*	12 (44.4)	8 (66.7)	20 (52.6)	
Birth weight (g)/median (1st–3rd quartile)	3000.0 (2600.0–3400.0)	3222.5 (3087.5–3555.0)	3100.0 (2700.0–3500.0)	0.342^a^
Consanguinity				
* None*	8 (30.8)	7 (58.3)	26 (66.7)	*0.033*^*b,x*^
* Present*	13 (50.0)	5 (41.7)	11 (28.2)	
* From the same village*	5 (19.2)	0 (0.0)	2 (5.1)	

^*^^a^Kruskal–Wallis test, ^b^Chi-square test/Fisher’s exact test

^x^IEI-related variants and undetected disease-causing variants *p* =.011 (Fisher’s exact)

*IEI* inborn errors of immunity, *NGS* next-generation sequencing, *TGP* targeted gene panels, *WES* whole-exome sequencing

Comparing the 28 patients with IEI diagnosed by NGS, 12 patients carrying clinically significant but non-IEI-related variants, and 39 patients undetected disease-causing variants (Table 2), certain differences in clinical presentation and laboratory parameters were observed. In all groups, infections were the predominant presenting complaint, especially pneumonia, bronchitis/bronchiolitis, and skin infections.

**Table 2:** Comparison of clinical presentation characteristics of the patients analyzed by TGP and WES according to variant characteristics

Clinical presentation	IEI-related variants (*n* = 28)	Non-IEI-related variants (*n* = 12)	Undetected disease-causing variants (*n* = 39)	*p*-value^a^
	*n* (%)	*n* (%)	*n* (%)	
Recurrent infection	24 (85.7)	11 (91.7)	31 (79.5)	0.776
* Pneumonia*	15 (53.6)	7 (53.6)	15 (38.5)	0.325
* Bronchitis/bronchiolitis*	9 (32.1)	5 (41.7)	17 (43.6)	0.637
* Otitis/sinusitis*	9 (32.1)	2 (16.7)	8 (20.5)	0.516
* Moniliasis (Candidiasis)*	2 (7.2)	0 (0.0)	3 (7.7)	0.814
* Diarrhea*	6 (21.4)	3 (25.0)	5 (12.8)	0.519
* Skin infection*	7 (25.0)	1 (8.3)	5 (12.8)	0.378
* Tuberculosis*	1 (3.6)	0 (0.0)	3 (7.7)	0.814
* Osteomyelitis*	1 (3.6)	0 (0.0)	2 (5.1)	1.000
* Meningitis/encephalitis*	1 (3.6)	1 (8.3)	5 (12.8)	0.501
Immunedysregulation	13 (46.4)	6 (50.0)	21 (53.8)	0.835
* Lymphoproliferation*	4 (14.3)	2 (16.7)	9 (23.1)	0.793
* Eczema/dermatitis*	5 (17.9)	2 (16.7)	3 (7.7)	0.431
* Asthma/allergic rhinitis*	3 (10.7)	3 (25.0)	6 (15.4)	0.478
* Hypothyroidism/thyroiditis*	2 (7.1)	1 (8.3)	1 (2.6)	0.478
* Lymphoma*	2 (7.1)	0 (0.0)	2 (5.1)	1.000
* Epilepsy*	0 (0.0)	1 (8.3)	5 (12.8)	0.122
* Autoimmune hepatitis*	1 (3.6)	0 (0.0)	2 (5.1)	1.000
* Eosinophilic esophagitis/gastropathy*	0 (0.0)	0 (0.0)	2 (5.1)	0.646
* Autoimmune hemolytic anemia*	0 (0.0)	0 (0.0)	1 (2.6)	1.000
* Inflammatory bowel disease*	1 (3.6)	0 (0.0)	0 (0.0)	0.506
*Allergic sensitivity*				
* Food allergy*	4 (14.3)	2 (16.7)	3 (7.7)	*0.023*^*x*^
* Inhalant allergen sensitivity*	0 (0.0)	3 (25.0)	1 (2.6)	
Growth and developmental delay	10 (35.7)	6 (50.0)	12 (30.8)	0.477
Dysmorphism	10 (35.7)	6 (50.0)	9 (23.1)	0.182

^a^Chi-square test/Fisher’s exact test

^x^IEI-related variants and non-IEI-related variants *p* =.020 (Fisher’s exact)

Non-IEI-related variants and undetected disease-causing variants *p* =.026 (Fisher’s exact)

In the IEI-related variant group, infectious manifestations were predominant (85.7%), with pneumonia (53.6%) and bronchitis/bronchiolitis (32.1%) being the most frequently observed. Immune dysregulation (46.4%) and growth retardation (35.7%) were also notable findings. Additionally, dysmorphic features (35.7%) were observed at a markedly higher frequency compared with the other groups. The clinical and genetic findings in patients with IEI-related and non-IEI-related tested with targeted gene panels are presented in Tables 3 and 4.

**Table 3:** Clinical and genetic findings in patients with IEI tested with *next-generation sequencing*

No	Sex	Genetic diagnosis age (year)	Clinical characteristics	Variant	Zygosity	ACMG classification	Gene	Inheritance
1	Female	2.03	Pneumonia, otitis, ataxia, neuromotor retardation	c.3746 + 1G > A	Homozygous	P	ATM	AR
2	Female	6.1	Ataxia, neuromotor retardation	c.77886G > A	Homozygous	P	ATM	AR
3	Male	3.49	Otitis, croup, growth retardation	c.1955 T > C	Hemizygous	P	BTK	XL
4	Female	9.46	Pneumonia/bronchitis, lymphoproliferation, lymphoma	c.137-1G > A(IVS1-1 > A)	Homozygous	LP	CD27	AR
5	Male	1.52	Abscess, otitis, growth retardation	c.347-1G > A	Hemizygous	P	CD40LG	XL
6	Male	2.72	Pneumonia	c.1141C > T (p.Arg381Ter)	Homozygous	P	CDCA7	AR
7	Male	8.91	Pneumonia/bronchitis, eczema, asthma, growth retardation	c.2421A > G (p.Arg167Trp)	Heterozygous	VUS	CFTR	AR
8	Female	2.44	Diarrhea, pneumonia, otitis, food allergy	c.328G > C (p.Asp110His) c.902A > G (p.Tyr302Cys)	Heterozygous Heterozygous	P VUS	CFTR	AR
9	Female	13.4	Diarrhea, inflammatory bowel disease, growth retardation	c.214 T > C	Homozygous	P	NCF1	AR
10	Male	10.8	Pneumonia, osteomyelitis, anal abscess, otitis, dysmorphism	c.1936A > T (p.lle646Phe)	Homozygous	VUS	DNMT3B	AR
11	Male	4.47	Recurrent upper respiratory tract infection, hypothyroidism	NM_000569.7 Exon 1–2 deletion, Exon 3 deletion, Exon 4–5 deletion	Homozygous	VUS	FCGR3A	AR
12	Female	14	Pneumonia/bronchitis	c.112C > T (p.Arg38Cys)	Heterozygous	LP	IRF2BP2	AD
13	Female	4.55	Pneumonia, otitis, asthma	c.442G > C (p.Glu148Gln)	Heterozygous	P	MEFV	AD/AR
14	Female	0.42	Diarrhea, sepsis, fever, growth retardation	c.478A > T (p.Lys160Ter)	Homozygous	LP	OTULIN	AD/AR
15	Male	2.09	Pneumonia, neuromotor retardation, hypothyroidism, growth retardation, dysmorphism	c.158C > A	Homozygous	LP	PAX1	AR
16	Male	3.64	Otitis, bronchitis, neuromotor retardation, dysmorphism	c.349G > A p.Ala117Thr	Homozygous	LP	PNP	AR
17	Female	1.23	Moniliasis, pneumonia, seborrheic dermatitis, neuromotor retardation, growth retardation	c.2 T > G	Homozygous	LP	PNP	AR
18	Female	10.8	Encephalitis, acute necrotizing encephalopathy	c.1795A > C	Heterozygous	VUS	RANBP2	AD
19	Male	1.35	Abscess, dysmorphism	c.2544_2546delinsCGA	Heterozygous	VUS	RTEL1	AD
20	Female	7.35	Peumonia, lobectomy, operated diaphragmatic hernia, onychomycosis on hand, developmental delay, dysmorphism	c.1907C > A (pSer636Tyr)	Heterozygous	P	STAT3	AD
21	Female	3.32	Moniliasis, otitis, bronchitis, eczema, seborrheic dermatitis, paronychia, food allergy, dysmorphism	c.1027G > T	Heterozygous	P	STAT3	AD
22	Female	1.63	Pneumonia/bronchitis, abscess, food allergy, diarrhea, sinusitis, otitis, bone fracture, rectovaginal fistula, dysmorphism	c.1145GZA (p.Arg382Gln)	Heterozygous	P	STAT3	AD
23	Male	14.8	Lymphoproliferation, lymphoma, growth retardation	c.1103del.p	Homozygous	P	STK4	AR
24	Male	6.65	Pneumonia/bronchitis, lymphoproliferation, asthma, tuberculosis	c.722A > C (p.His241Pro)	Homozygous	LP	TNFSF13	AR
25	Male	2.91	Diarrhea, bronchitis, food allergy	c.3039A > G	Homozygous	VUS	TTC37	AR
26	Male	2.54	Diarrhea, pneumonia, growth retardation	c.4507C > T (p.Arg1503Cys) c.2170 T > C (p.Cys724Arg)	Heterozygous Heterozygous	VUS VUS	TTC37	AR
27	Male	1.55	Diarrhea, pneumonia/bronchitis, growth retardation, dysmorphism	c.531del	Homozygous	P	USB1	AR
28	Male	17.6	Severe viral infection, hepatic fibrosis, dysmorphism, left ventricular hypertrophy, recurrent pneumothorax	c.3228C > A (p.Cys1076)	Homozygous	LP	ZNFX1	AR

**Table 4:** Clinical and genetic findings in patients with non-IEI-related tested with next-generation sequencing

No	Sex	Genetic diagnosis age (year)	Clinical characteristics	Variant	Zygosity	ACMG classification	Gene	Inheritance
1	Male	7.82	Otitis, pneumonia	c.3025G > C	Hemizygous	VUS	OFD1	XL
2	Male	0.85	Sepsis, eczema, lymphoproliferation, dysmorphism	c.848 T > C	Heterozygous	LP	CASR	AD/AR
3	Female	2.02	Diarrhea, inflammatory bowel disease, dysmorphism	c.503A > G p.Lys168Arg	Homozygous	LP	PMM2	AR
4	Female	0.36	Ichthyosis, eczema, sepsis	c.372 + 1G > T IVS4 + 1G > T	Homozygous	P	DSG1	AR
5	Female	2.18	Otitis, pneumonia, dysmorphism	c.1811A > C	Heterozygous	LP	ZBTB20	AD
6	Female	1.46	Sepsis, eczema, gastrointestinal bleeding	c.469A > G p.Asn157Asp	Homozygous	LP	SCL27A4	AR
7	Male	0.5	Sepsis, pneumonia, dysmorphism	c.931G > A	Homozygous	P	GLB1	AR
8	Male	13.97	Pneumonia, asthma	c.1060G > A p.Asp354Asn	Heterozygous	VUS	FOXJ1	AD
9	Female	1.52	Pneumonia, epilepsy, dysmorphism	c.1358A > G p.Glu453Gly	Heterozygous	LP	PIK3CA	OD
10	Female	15.86	Inflammatory bowel disease, recurrent fever	c.2657G > A p.Arg886Gln	Heterozygous	LP	RET	OD
11	Female	4.75	Pneumonia, asthma	c.11691-19delinsTG c.11782C > G	Homoziygous Heterozygous	VUS	DNAH11	OR
12	Male	0.74	Meningitis, dysmorphism	c.79 + 1G > A	Homoziygous	P	RSPO4	AR

Among patients carrying non-IEI-related variants, the presence of infections (91.7%) was comparable to that of the IEI group. Pneumonia (53.6%) and bronchitis/bronchiolitis (41.7%) were also common in this group. Growth retardation (50.0%) and dysmorphic features (50.0%) were observed more frequently than in the IEI group. Moreover, sensitization to inhalant allergens (25.0%) was a distinguishing feature specific to this group.

The group in which immune dysregulation (53.8%) and lymphoproliferation (23.1%) were most frequently observed was undetected disease-causing variants. Although the overall infection rate (79.5%) was relatively lower, bronchitis/bronchiolitis (43.6%) and pneumonia (38.5%) remained common. Neurological complications such as epilepsy (12.8%) and meningitis/encephalitis (12.8%) were observed more frequently in this group. No statistically significant differences were found among the three groups regarding the frequency of infections or manifestations of immune dysregulation, including lymphoproliferation, eczema, and autoimmune diseases (*p* > 0.05). Regarding allergic manifestations, food allergy was more prevalent in the IEI group (14.3%), whereas sensitization to inhalant allergens was more frequent in the non-IEI group (25.0%). In contrast, the majority of patients in all groups had no evidence of allergic sensitization (85.7% in the IEI-related variant, 58.3% in the non-IEI-related variant, and 89.5% in the undetected disease-causing variants). Overall, the profiles of allergic sensitization differed significantly among the groups (*p* = 0.023).

## Discussion

In this study, demographic, clinical, and laboratory findings were comparatively evaluated with genetic analysis results in a heterogeneous pediatric population referred for genetic testing due to suspected IEI. The diagnostic contribution of NGS-based approaches was assessed, and the clinical and phenotypic characteristics of the subgroup with clinically significant non-IEI mutations were analyzed in detail. Our findings strongly emphasize that the diagnostic process should not remain confined to an immunology-centered approach; instead, dysmorphic, neurodevelopmental, respiratory, and dermatologic features should be assessed in an integrated manner. The genetic heterogeneity of IEI and diagnostic delays in atypical cases can lead to substantial morbidity and mortality. Establishing a definitive genetic diagnosis is therefore crucial for optimal patient management.

In 2016, Cairo University published its 5-year experience, reporting that a confirmed genetic diagnosis was achieved in only 22.26% of clinically diagnosed patients [10]. In the same country, the application of advanced genetic techniques has substantially improved diagnostic yield, reaching over 50% in a study, reflecting the significant progress in genetic testing since 2016 [4]. With the increasing use of genetic testing and the expansion of collaborative efforts among different centers, a recent multicenter cohort study conducted in 2024 in Türkiye, including 303 patients with IEI, reported a diagnostic yield of 41.1% using WES [11]. In our cohort, 35.4% of 79 patients undergoing NGS had IEI-related variants, while 15.2% carried clinically significant but non-IEI-related variants, a group of particular interest due to their potential to explain overlapping or mimicking phenotypes. In contrast, 49.4% of patients undetected disease-causing variants. These findings indicate that approximately half of the patients could not be genetically diagnosed. This emphasizes both the diagnostic limitations of currently available genetic tests and the persistent uncertainty in elucidating the underlying etiology of many IEI cases. The results further suggest a potential role for structural variants, intronic mutations, epigenetic alterations, and defects involving as-yet-undiscovered genes. They also highlight the importance of integrating advanced genomic technologies (such as whole-genome sequencing and RNA sequencing) into clinical practice to improve diagnostic yield and expand our understanding of the genetic spectrum of IEI and related disorders.

According to studies conducted in the Middle East and North Africa region, most cases (65.2%) exhibit autosomal recessive defects, with consanguinity reported in 60.5% of patients and a positive family history of IEI in 27.3% [12]. In our study, among the 28 patients diagnosed with IEI by NGS, 20 (71.4%) demonstrated autosomal recessive inheritance. Consanguinity between parents was observed in 50% of these patients, and 19.2% shared the same village of origin. Similarly, a proportion of patients carrying clinically significant but non-IEI-related genetic variants also had a family history of consanguinity. This observation indicates that consanguinity may play a role not only in the occurrence of IEI but also in other clinically relevant monogenic disorders. Therefore, careful evaluation of familial background and ethnic origin is essential in the diagnostic and genetic counseling processes for both IEI and non-IEI-related genetic diseases.

In our cohort, the most common clinical features in patients diagnosed with IEI were recurrent pneumonia, skin infections, and otitis/sinusitis with bronchiolitis. Similarly, Verma et al. reported that recurrent or persistent lower respiratory tract infections were the most frequent presentation among 27 pediatric IEI cases [13]. In contrast, Gupta et al., in a study of 120 children from northern India, found that lower respiratory infections, gastrointestinal symptoms such as diarrhea, and growth retardation were the primary indicators of IEI [14]. Such differences may be attributed to regional variations, differing levels of physician awareness, or population-specific factors such as consanguinity.

Recent studies have demonstrated that patients with IEI may present not only with increased susceptibility to infections but also with inflammatory bowel disease, autoimmunity, lymphoproliferative disorders, malignancies, neurodevelopmental delay, allergic diseases, eczema, growth retardation, and various neurological or gastrointestinal manifestations [15]. In our study, clinical findings such as eczema/dermatitis, asthma/allergic rhinitis, lymphoproliferation, lymphoma, and autoimmune thyroiditis were observed in both the IEI group and the group carrying clinically significant but non-IEI-related variants; however, these associations did not reach statistical significance. In our data, a high frequency of infections was also observed among patients with non-IEI-related variants. These patients exhibited a heterogeneous spectrum of disorders, including PCD and several rare genetic syndromes; notably, PCD is clinically difficult to distinguish from IEI and other conditions causing recurrent oto-sinopulmonary infections [16]. From a clinical perspective, these findings highlight that in patients presenting with recurrent infections suggestive of immunodeficiency, underlying ciliary, metabolic, neurocutaneous, or skeletal disorders should also be considered.

IEI may present with atopic phenotypes driven by impaired epithelial barrier integrity, dysregulated type 2 inflammation, and altered immune tolerance. Several clinical features, such as congenital ichthyosis, skeletal abnormalities, neurodevelopmental delay, diarrhea, endocrinopathy, bleeding, and/or failure to thrive, may be IEI-related with atopic phenotypes [17]. In our study; the allergic sensitization profiles were also noteworthy: sensitization to inhalant allergens was more prominent in the non-IEI-related group (25.0%), whereas food allergy predominated in the IEI group (14.3%). This finding suggests that aeroallergen sensitization may be more prevalent in syndromic conditions accompanied by epithelial barrier dysfunction, respiratory motility defects (e.g., PCD), or congenital anomalies. In contrast, food allergy during early childhood may shape the clinical phenotype in certain immune dysregulation disorders.

Several genetic and systemic conditions have been shown to mimic inborn errors of immunity, leading patients to present with recurrent or severe infections and immune abnormalities despite the absence of a primary immune defect. In a large cohort of children evaluated for suspected IEI, genetic testing revealed alternative non-IEI diagnoses in a subset of patients, including metabolic disorders such as Wolman and Gaucher disease, osteopetrosis, congenital vitamin B12 deficiency, and myelodysplastic syndrome, highlighting that diverse non-immunological conditions may clinically resemble IEI presentations [18]. Similarly, metabolic disorders such as methylmalonic aciduria have been reported to manifest with recurrent severe infections in early life and to be initially investigated as primary immunodeficiency, while immune abnormalities in these cases are likely secondary to underlying metabolic dysfunction [19]. These observations underscore the importance of carefully distinguishing true inborn errors of immunity from phenocopies and mimicking conditions, and emphasize that genetic findings should always be interpreted in conjunction with clinical presentation, immunological evaluation, functional data, and longitudinal follow-up to avoid misdiagnosis and inappropriate management.

In a previously analyzed cohort, immune dysregulation disorders were most common (19%), followed by antibody deficiencies (14%) and phagocytic defects (13.4%) [18]. Similarly, a study from the United Arab Emirates found that combined immunodeficiencies with syndromic features were most prevalent (38.3%), followed by cellular and humoral immunodeficiencies (20.4%) and antibody deficiencies (16%) [15]. In our study, the most frequently identified category of IEI was combined immunodeficiency with syndromic features. This was followed, in order of frequency, by disorders of phagocyte number and function, intrinsic and innate immunity defects, antibody deficiencies, and combined immunodeficiencies. This distribution suggests that patients referred for genetic evaluation, with severe or syndromic phenotypes are more likely to achieve a definitive molecular diagnosis. Dysmorphic features were also observed in patients carrying clinically significant but non-IEI-related variants, further highlighting the phenotypic overlap between syndromic and immunologic disorders.

A notable finding of our study was that some patients remained without a molecular diagnosis or carried variants unrelated to their clinical features, despite comprehensive genetic testing. This underscores the limitations of current genetic panels and WES in detecting all forms of IEI and highlights the need for discovering new genes and conducting functional validation studies [20]. Additionally, several detected variants were classified as variants of unknown significance, whose clinical impact remains unclear.

## Conclusion

Our study provides evidence supporting the critical role of genetic testing in the differential diagnosis of IEI, particularly in patients with mixed or atypical IEI phenotypes. Despite advances in NGS technologies, a subset of patients remains undiagnosed or carries variants unrelated to immune deficiency, underscoring the continued need for novel gene discovery. This work also emphasizes that the scope of genetic evaluation should extend beyond classical IEI, as clinically significant non-IEI-related variants can explain overlapping or syndromic phenotypes that mimic immunodeficiency. A multidisciplinary approach integrating clinical, laboratory, and genetic data is essential for achieving early and accurate diagnosis, guiding patient management, and informing genetic counseling.

In summary, multidisciplinary approach combined with comprehensive genetic evaluation is crucial for the early diagnosis and effective management of both IEI and non-IEI disorders, as it not only refines diagnostic precision within the immunodeficiency spectrum but also broadens the clinical perspective by uncovering non-IEI conditions, ultimately optimizing outcomes and ensuring individualized care for patients presenting with mixed IEI phenotypes.

## Data Availability

The datasets generated and analyzed during the current study contain sensitive clinical and genetic information and are therefore not publicly available due to patient privacy and ethical restrictions.

## Abbreviations

ACMG/AMP: American College of Medical Genetics and Genomics/Association for Molecular Pathology
BTK: Bruton tyrosine kinase
FISH: Fluorescence in situ hybridization
HGMD: Human gene mutation database
IEI: Inborn errors of immunity
Ig: Immunoglobulin
IUIS: International Union of Immunological Societies
NGS: Next-generation sequencing
PCD: Primary ciliary dyskinesia
PNP: Purine nucleoside phosphorylase
STAT3: Signal Transducer and Activator of Transcription 3
TGP: Targeted gene panels
WES: Whole-exome sequencing
VUS: Variants of uncertain significance
